# Flaxseed oil fraction reverses cardiac remodeling at a molecular level: improves cardiac function, decreases apoptosis, and suppresses miRNA-29b and miRNA 1 gene expression

**DOI:** 10.1186/s12906-023-04319-8

**Published:** 2024-01-02

**Authors:** Sylvia A. Boshra, Jilan A. Nazeam, Ahmed Esmat

**Affiliations:** 1 Biochemistry Department, Faculty of Pharmacy, October 6 University, 6 of October City, Giza, 12585 Egypt; 2 Pharmacognosy Department, Faculty of Pharmacy, October 6 University, 6 of October City, Giza, 12585 Egypt; 3 Department of Pharmacology and Toxicology, Faculty of Pharmacy, Ain Shams University, Cairo, 11566 Egypt

**Keywords:** Flaxseed oil, miRNA-1, ISO, CVD, MMP9, cTnI

## Abstract

Flaxseed is an ancient commercial oil that historically has been used as a functional food to lower cholesterol levels. However, despite its longstanding treatment, there is currently a lack of scientific evidence to support its role in the management of cardiac remodeling. This study aimed to address this gap in knowledge by examining the molecular mechanism of standardized flaxseed oil in restoring cardiac remodeling in the heart toxicity vivo model. The oil fraction was purified, and the major components were standardized by qualitative and quantitative analysis. In vivo experimental design was conducted using isoproterenol ISO (85 mg/kg) twice subcutaneously within 24 h between each dose. The rats were treated with flaxseed oil fraction (100 mg/kg orally) and the same dose was used for omega 3 supplement as a positive control group. The GC-MS analysis revealed that α-linolenic acid (24.6%), oleic acid (10.5%), glycerol oleate (9.0%) and 2,3-dihydroxypropyl elaidate (7%) are the major components of oil fraction. Physicochemical analysis indicated that the acidity percentage, saponification, peroxide, and iodine values were 0.43, 188.57, 1.22, and 122.34 respectively. As compared with healthy control, ISO group-induced changes in functional cardiac parameters. After 28-day pretreatment with flaxseed oil, the results indicated an improvement in cardiac function, a decrease in apoptosis, and simultaneous prevention of myocardial fibrosis. The plasma levels of BNP, NT-pro-BNP, endothelin-1, Lp-PLA2, and MMP2, and cTnI and cTn were significantly diminished, while a higher plasma level of Topo 2B was observed. Additionally, miRNA − 1 and 29b were significantly downregulated. These findings provide novel insight into the mechanism of flaxseed oil in restoring cardiac remodeling and support its future application as a cardioprotective against heart diseases.

**Supplementary Information:**

The online version contains supplementary material available at 10.1186/s12906-023-04319-8.

## Introduction

Cardiovascular disease (CVDs) is a leading cause of disability and premature mortality throughout the world [1]. According to WHO estimates, 17.9 million people died from CVDs in 2019, accounting for 32% of all fatalities. The prevalent case was shifted from 271 million cases in 1990 to 523 million cases in 2019. Moreover, the global trend of years lived with a disability doubled from 17.7 million to 34.4 million over that period [2]. Hence, there is an urgent need to focus on adopting existing low-cost public health programs to minimize disability and premature death from CVD throughout the lifetime [3].

Fibrosis is a significant global healthcare burden and a major contributor to organ failure [4]. Pyroptosis, is an innate immune response and form of programmed cell death, that is characterized by the release of pro-inflammatory cytokines, which activate inflammasomes, myofibroblasts, and ultimately lead to fibrosis [5]. Consequently, there is growing evidence indicating that pyroptosis triggers the development of fibrosis [6]. Additionally, frequent pyroptosis has been linked to cardiac fibrosis, cardiomyocyte death, myocardial dysfunction, cardiac hypertrophy, and cardiac remodeling [7]. According to the literature, targeting pyroptosis has the potential as a therapeutic approach for reversing tissue injury and cardiac remodeling in heart failure disease [8].

In the last decade, functional foods global interests have increased enormously as they play an outstanding role in reducing the risk of chronic disease [9]. *Linum usitatissimum* L. (flaxseed or linseed) is one of the oldest crops, that has been cultivated in Egypt and Samaria since at least 5000 BC, and the ancient Egyptians utilized plants in medicine and food [10]. It reaches back to prehistoric times and directly refers to historical significance and wide applications, the generic name *Linum* derives from the Celtic word *lin*, which means thread, and the species name *usitatissimum*, which means very useful [11]. The geographical origin of the plant has been attributed to the Mediterranean and Southwest Asia [12]. According to archeological evidence, flax was initially used for fiber and continues to be widely grown for oil [13].

The flax-based products represented 1.8 and 8.7 million tons, worldwide in 2011 and 2016 [14, 15]. It has been reported to assist in improving human health and alleviating symptoms associated with a wide range of human ailments, such as cardiovascular, gastrointestinal, and neural disorders, atherosclerosis, and hypercholesterolemia [16–18]. Clinical studies have revealed that flaxseed can lower serum total and low-density lipoprotein cholesterol, inhibit inflammation markers, and raise serum levels of eicosapentaenoic acid [19]. In young healthy adults, the plasma LDL cholesterol was lowered by 8% after the consumption of 50 g flaxseed/day for four weeks [20]. Moreover, in a clinical trial for 50 hypercholesterolemic men, the C-reactive protein and serum amyloid levels were reduced after daily consumption of one tablespoon of flax oil for 12 weeks [21, 22]. According to a previous study, flaxseed oil regulated lipid metabolism-related genes (HMGCR, PPARα, and SREBPs) and improved atherosclerosis in heart failure disease (HFD) induced rats [23].

Flaxseed oil is characterized by the abundance of linolenic acid secondary metabolites that are well known for possessing cardioprotective effects [24, 25]. The current study advocates a potential pleiotropic effect of flaxseed oil beyond the more conventional cholesterol-lowering actions of most cardiovascular agents. Hence, the study aims to explore the cardioprotective molecular mechanism and genetic modulation effect of flaxseed oil to restore cardiac remodeling in order to verify the popular and clinical claim of its use against cardiac disorder.

## Materials and methods

### Extraction and fractionation of flaxseed oil

The flaxseed (500 g) was grounded to about 0.8 mm particle size (18–20 mesh) and macerated with dichloromethane: methanol (1:1) for 72 h [26–35]. The filtrate was concentrated using a rotary evaporator and 250 ml oil was collected and dried over anhydrous sodium sulfate. The fixed oil was fractionated over the Diaion column (5 × 50 cm) and the methanol fraction was transferred to amber-colored vials, sealed, and stored in a refrigerator until required.

### Analysis of essential oils (EOs)

The fixed oil fraction was analyzed by GC-MS (Shimadzu GCMS-QP 2010, Koyoto, Japan) equipped with Rtx-5MS capillary column (30 m length × 0.25 mm i.d. × 0.25 μm film thickness) (Restek, Bellefonte, PA, USA). The oven temperature was kept at 50 ^◦^C for 2 min (isothermal) and programmed to 300 ^◦^C at 5 ^◦^C/min and kept constant at 300 ^◦^C for 10 min (isothermal); the injector temperature was 280 ^◦^C. Helium was used as a carrier gas with a constant flow rate set at 1.37 mL/min. Diluted samples (1% v/v) were injected with a split ratio of 30:1, and the injected volume was 1 µL. Ion source temperature: 300 ^◦^C; EI mode: 70 eV; scan range: 35–500 amu. Each sample was analyzed in triplicate. The mass spectrum of every chemical constituent was compared with the corresponding reported spectrum (in NIST, Wiley Mass Spectral Database − 1995, and ADAMS-2007 libraries) for GC-MS and published references. Identification of compounds was also confirmed by comparing their retention indices (RI) relative to n-alkanes (C8-C20) with reported values in the literature including Adamís library.

### Specific gravity analysis

The specific gravity (SG) of the oil samples was determined by using a pycnometer [36] and its specific gravity was calculated by the following formula:

$${\rm{Specific}}\,{\rm{gravity}}\,{\rm{ = }}\,{\rm{density}}\,{\rm{of}}\,{\rm{oil/density}}\,{\rm{of}}\,{\rm{water}}$$

### Iodine value analysis

The iodine value (IV) of the tested samples was determined using the Hanus method according to the method described in [36]. Briefly, about 0.25 g of the tested sample was dissolved in 20 ml chloroform. 25 ml Hanus iodine (13.2 g pure I_2_ in 1 L CH_3_COOH) solution was added and let stand 30 min in dark. 10 ml 15% KI solution was placed, shaken thoroughly then 100 ml freshly boiled and cooled H_2_O was added. I_2_ was titrated with 0.10 N sodium thiosulphate solution with constant shaking until the yellow solution turned almost colorless. A few drops of 1% starch solution were used and titration was continued until the blue entirely disappeared (shaking vigorously to release all iodine from CHCl_3_). A blank was performed omitting the oil, and the iodine value was calculated as grams of iodine per 100 g of oil.

$$Iodine\,value\, = \,\left( {B\,-\,S} \right)\, \times \,N\, \times \,12.69/w$$

Where:

B = ml Na_2_S_2_O_3_ solution required for blank

S = ml Na_2_S_2_O_3_ solution required for test sample

N = normality of Na_2_S_2_O_3_ solution

W = weight of sample in g

### Peroxide value analysis

The peroxide value (PV) was determined according to the [36]. Two g of tested oil were weighed in a flask with a ground-glass cap. 250–300, 10 ml of chloroform was added and shacked for 10 min, 15 ml of glacial acetic acid, and 2 g of sodium bicarbonate NaHCO_3_ was used after stirring, 1 ml of a saturated solution of KI was placed and shacked for 1 min and kept in dark place for 5 min, immediately. After the specified time, 75 ml of distilled water, and then 0.5 ml of starch solution were used, the resulting solution was titrated with a solution of 0.002 N Na_2_S_2_O_3_ to the disappearance of the blue color, and the blank was carried on the same procedures but without sample. A peroxide value of the tested oil is given by the equation:

$$PV\, = \,\left[ {\left( {V1 - V2} \right)\,*\,N\,*\,1000} \right]\,/\,m\left[ {meq.{O_2}/kg} \right]$$

Where: *V1* = volume of sodium thiosulfate solution consumed in the titration sample of the [ml], *V2* = volume of sodium thiosulfate consumed in the titration of the blank [ml], *N* = the normality of sodium thiosulfate, *m* = weight of fat taken to denote [g].

### Saponification value

The saponification value of the tested oil samples was determined according to the method described in [36]. Briefly, 5 g of the tested sample was weighed into a 250–300 ml conical flask, and 50 ml alcoholic KOH solution (35–40 g KOH was dissolved in 20 ml water and diluted to one liter with alcohol, 95%) was placed. The flask was connected with an air condenser, boiled until fat was completely saponified (~ 30 min), cooled, and titrated with 0.5 M HCl using phenolphthalein. The saponification value was calculated by the following formula:

$$Saponification\,value\, = \,28.05\,\left( {B - S} \right)/W\,mg/g$$

Where B mL HCl is required for blank, S mL HCl is required for the test sample, and W weight of the sample in g.

### Acid value analysis

The method used for the analysis of acid value (AV) was adapted from [37]. A mixture of absolute ethanol and diethyl ether (1:1 v/v) was carefully neutralized with 0.10 N potassium hydroxide solution using 1% phenolphthalein indicator. Five g of the tested samples were dissolved in 50 ml neutralized ethanol-diethyl ether solvent and titrated with 0.10 M potassium hydroxide with constant shaking until a pink color persisted for 15 s. Free fatty acids were calculated as % of oleic acid.

$$FFA\,\% \,as\,oleic\,acid\, = \,S\, \times \,0.0282\, \times \,100\,/\,w$$

Where: S = titration (ml), w = weight of the oil (g).

### In-vivo experimental design

Thirty-six male Sprague-Dawley rats, each weighing 160 ± 10 g, were provided from the National Cancer Institute, Cairo University. Animals were housed in the vivarium of October 6 University under a 12-hour light-dark cycle and standard humidity. Rats were given unlimited access to regular pellets and water. The study was carried out according to the guidelines of October 6 University and approved – in advance- by the Ethics Committee of the Faculty of Applied Medical Science (protocol code: 20,220,301). Isoproterenol (ISO) is a synthetic catecholamine and b-adrenergic agonist that infarct-like necrosis of the heart muscle and oxidative stress [38]. Through autooxidation, it produces highly cytotoxic free radicals that speed up the peroxidation of membrane phospholipids and seriously damage the heart membrane [39]. Animals were randomly divided into four groups (8 rats each). Group I served as a control for 30 days, while group II received isoproterenol (ISO) 85 mg/kg, intraperitoneal (IP) on days 29 and 30 of the study. Groups III and IV were pretreated with flaxseed and omega-3 oils; respectively, (100 mg/kg/day) orally for 30 days accompanied by ISO (85 mg/kg, ip) on days 29 and 30. According to Gorriti et al., LD_50_ of flaxseed oil is above 37 g/kg of body weight, the tested dose of flaxseed was selected based on its safety for animals and the same dose was used for omega-3 capsule (alpha-linolenic acid, eicosapentaenoic acid and docosahexaenoic acid) as a standard drug [40]. After 24 h of the last ISO dose, animals were anesthetized using a ketamine/xylazine mixture and blood was collected on EDTA tubes to separate plasma samples. Rats were then sacrificed with cardiac tissues dissected and kept for real-time polymerase chain reaction (RT-PCR) based on the instructions of RNeasy Mini Kit (Qiagen, Hilden, Germany).

Assessing the plasma levels of brain natriuretic peptide (BNP), atrial natriuretic peptide (NT-pro-BNP), cardiac troponin I (cTnI), cardiac troponin T (cTnT), and endothelin-1 were carried out utilizing ELISA kits purchased from Abcam (Cambridge, UK), with catalog numbers: ab108816, ab263877, ab246529, ab223860, ab133030; respectively. In addition, topoisomerase 2 beta (Topo 2B), lipoprotein-associated phospholipase A2 (Lp-PLA2), and matrix metalloproteinase 9 (MMP9) were determined using ELISA kits obtained from Mybiosource (San Diego, CA, USA) with catalog numbers: MBS078781, MBS3809830, and MBS3809207, correspondingly.

### Gene expression of miRNA-1 and miRNA-29b by quantitative real-time PCR

Following the manufacturer’s recommendations, total RNA was isolated from cardiac tissues using an RNeasy Mini Kit (Qiagen, Hilden, Germany), and then (10–15 ng) of the obtained RNA was subjected to real-time quantitative PCR testing. A two-step RT-PCR was used to quantify gene expression. Quantitative real-time PCR was used to measure the levels of miRNA-1 and miRNA29b.PCR buffer, 1.5 mM MgCl2, 0.2 mM of each dNTP, and 0.4 M of specific primers made up the PCR reaction mixture (Table 1). In 50 µl of the single-plex reaction mixture, assays were carried out. Forty cycles of 95 ^o^ C for 15 s and 60 ^o^ C for 1 min each made up the reaction conditions, which also included a pre-incubation at 50 ^o^ C for 2 min and 95 ^o^ C for 10 min. The measurements were automatically taken down. Data from quantitative RT-PCR are displayed as a percentage of the control. The internal check was done using U6 mRNA.

**Table 1:** Primers used in real-time PCR.

Gene	Primer sequence	Amplicon Size
miRNA1	F: 5′-ACACAGAGAGGGCTCCGGCA-3′ R:5′-ACACGACCGTCCACCAACGC-3′	342 bp
miRNA 29b	F: 5′-GCTGGTTTCATATGGTGG-3′ R: 5′-GAACATGTCTGCGTATCTC-3′	141 bp
U6 (internal control for qRT-PCR)	F: ‘5- GCTTCGGCAGCACATATACTAAAAT − 3′; R: 5′- CGCTTCACGAATTTGCGTGTCAT − 3′.	84 bp

### Statistical analysis

Results are presented as mean ± SD of 8 replicates. Analyses were executed using one-way ANOVA followed by Tukey’s as a post-*hoc* test. The level of significance was set at *p* < 0.01. GraphPad Prism software version 8 (La Jolla, CA, USA) was utilized to finalize all statistical analyses and sketch graphs.

## Results

### Chemical analysis of purified oil

Physicochemical analysis revealed the values of various parameters examined. The acid, saponification, peroxide, and iodine values were 0.43, 188.57, 1.22, and 122.34 respectively (Table 2). Characterization and standardization of purified oil fraction by GC-MS analysis revealed the presence of 83 compounds (Supplementary file Table I), the major constituents were alpha-linolenic acid (24.69%), oleic acid (10.57%), glycerol oleate (9.04%), 2,3-dihydroxypropyl elaidate (7.05%), n-propyl linolenate (6.05%), elaidic acid (4.45%), and palmitic acid (4.02%) (Table 3).

**Table 2:** Physicochemical analysis of flaxseed oil fraction

Parameters	Value ± STDEV
Specific gravity	0.921 ± 0.002
Acidity (%)	0.43 ± 0.03
Peroxide value (meq/kg)	1.22 ± 0.08
Iodine value (g/100 g)	122.34 ± 1.52
Saponification value (mg/g)	188.57 ± 2.16

**Table 3:** GC-MS of purified flaxseed oil fraction

Peak	R. Time	Area%	Name	Base m/z
19	34.493	4.02	Palmitic acid	73.10
23	37.053	4.45	Elaidic acid	97.15
25	37.995	24.69	Alpha-linolenic acid	79.10
26	38.095	10.57	Oleic Acid	83.15
41	41.689	6.05	n-Propyl linolenate	79.10
42	41.771	7.05	2,3-Dihydroxypropyl elaidate	83.10
55	46.641	9.04	Glycerol oleate	98.15

### In vivo study

**Fig. 1:**
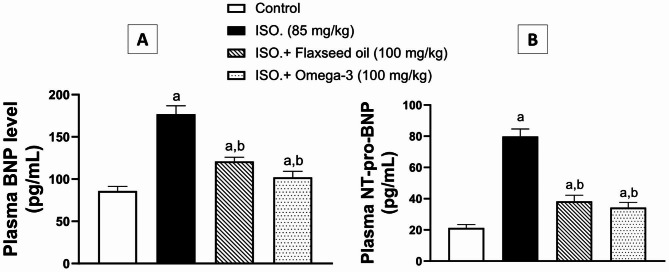
Effect of 28-day pretreatment with flaxseed oil on: [**A**] BNP and [**B**] NT-pro-BNP in isoproterenol-induced cardiac remodeling in rats. Data are represented as mean ± S.D. n = 8. Statistical analysis was performed utilizing one-way ANOVA followed by Tukey’s post-hoc test. The level of significance was set at p < 0.01. (**a**) is significantly different from the corresponding control group; (**b**) is significantly different from the corresponding isoproterenol-challenged group. ISO: isoproterenol; BNP: Brain Natriuretic Peptide, NT-pro-BNP: N Terminal of pro-B type Brain Natriuretic Peptide

The effects of flaxseed pretreatment on plasma levels of BNP and NT-pro-BNP are illustrated in Fig. 1. Briefly, ISO administration (group II) resulted in a significant (p < 0.01) two-fold increase in BNP level (Fig. 1A) and about four-fold increase (*p* < 0.01) of NT-pro-BNP (Fig. 1B), in comparison to the corresponding control group. Animals pretreated with flaxseed for 28 days (group III) showed significant decreases of BNP and NT-pro-BNP levels by about 32% and 52%, respectively (p < 0.01), as compared to the corresponding ISO group (II). Furthermore, pretreatment with the standard omega-3 (group IV) caused significant declines of BNP and NT-pro-BNP levels by about 42% and 57%, respectively (p < 0.01), as compared to the ISO group (II).

**Fig. 2:**
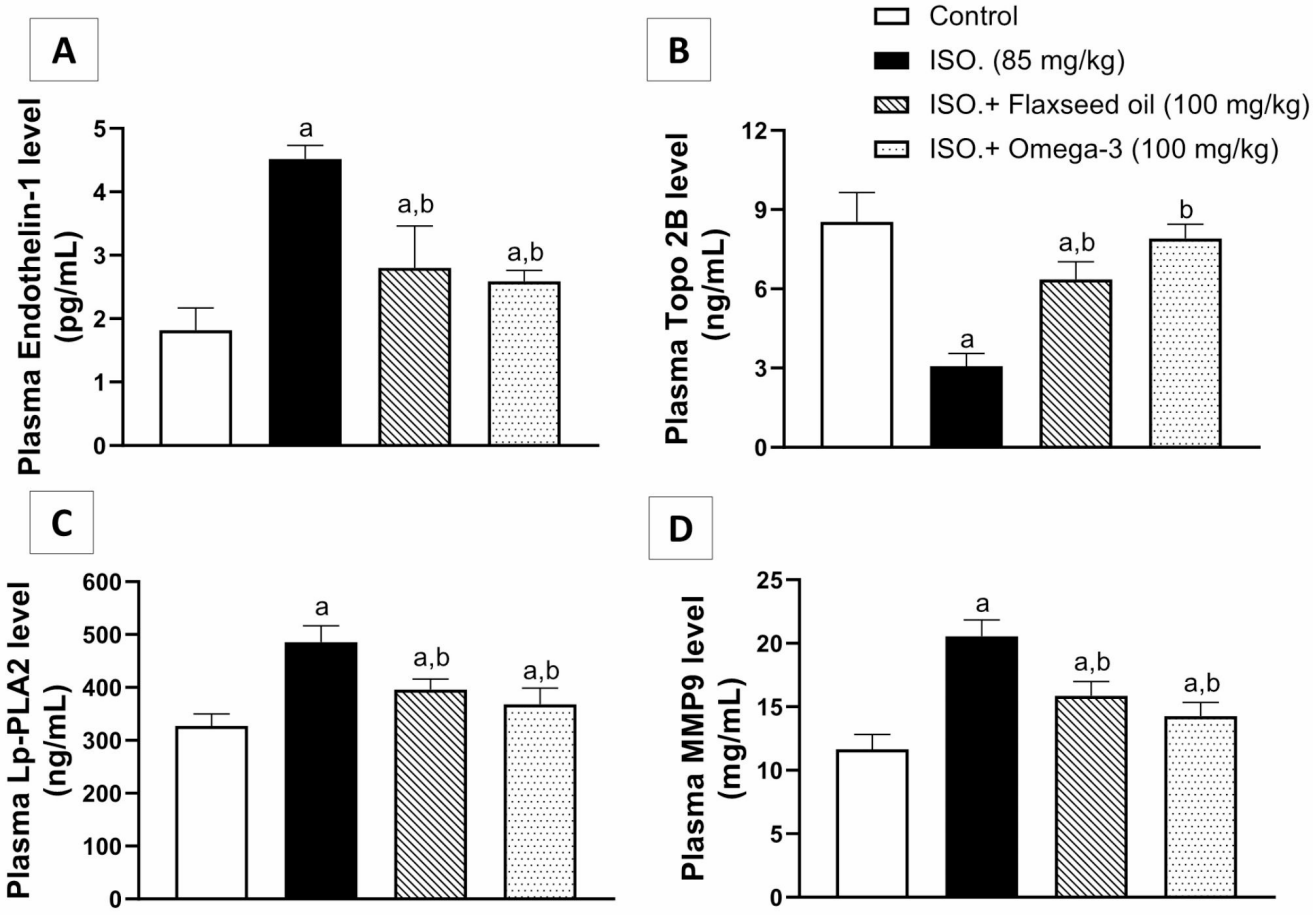
Effect of 28-day pretreatment with flaxseed oil on: [**A**] Endothelin-1; [**B**] Topo 2B; [**C**] Lp-PLA2 and [**D**] MMP9 in isoproterenol-induced cardiac remodeling in rats. Data are represented as mean ± S.D. n = 8. Statistical analysis was performed utilizing one-way ANOVA followed by Tukey’s post-hoc test. The level of significance was set at p < 0.01. (**a**) is significantly different from the corresponding control group; (**b**) is significantly different from the corresponding isoproterenol-challenged group. Topo 2B: Topoisomerase 2 beta; Lp-PLA2: Lipoprotein-Associated Phospholipase A2, MMP9: N Matrix metalloproteinase 9

A similar pattern of activity was observed with assessing the flaxseed effect on plasma levels of endothelin-1, Lp-PLA2, and MMP9. Compared to the corresponding controls, ISO administration (group II) induced significant rises (*p* < 0.01) in plasma endothelin-1, Lp-PLA2, and MMP9 by about 150%, 49%, and 79%, respectively, as displayed in Fig. (2 A, 2 C & 2D). On the contrary, it significantly (p < 0.01) reduced plasma Topo 2B by ~ 64%, as shown in Fig. (2B). These effects were all ameliorated by flaxseed pretreatment (group III), where it significantly (p < 0.01) lowered plasma endothelin-1, Lp-PLA2, and MMP9 levels by 38%, 19%, and 23%, respectively (Fig. 2A C & 2D), while raised (*p* < 0.01) the level of Topo 2B by about two folds (Fig. 2B), compared to ISO group (II). As regards the standard omega-3 pretreatment (group IV), endothelin-1, Lp-PLA2, and MMP9 levels were considerably decreased (*p* < 0.01) by ~ 43%, 24%, and 31%, respectively, while Topo 2B level was significantly increased by more than two folds (*p* < 0.01), in comparison to ISO group (II).

**Fig. 3:**
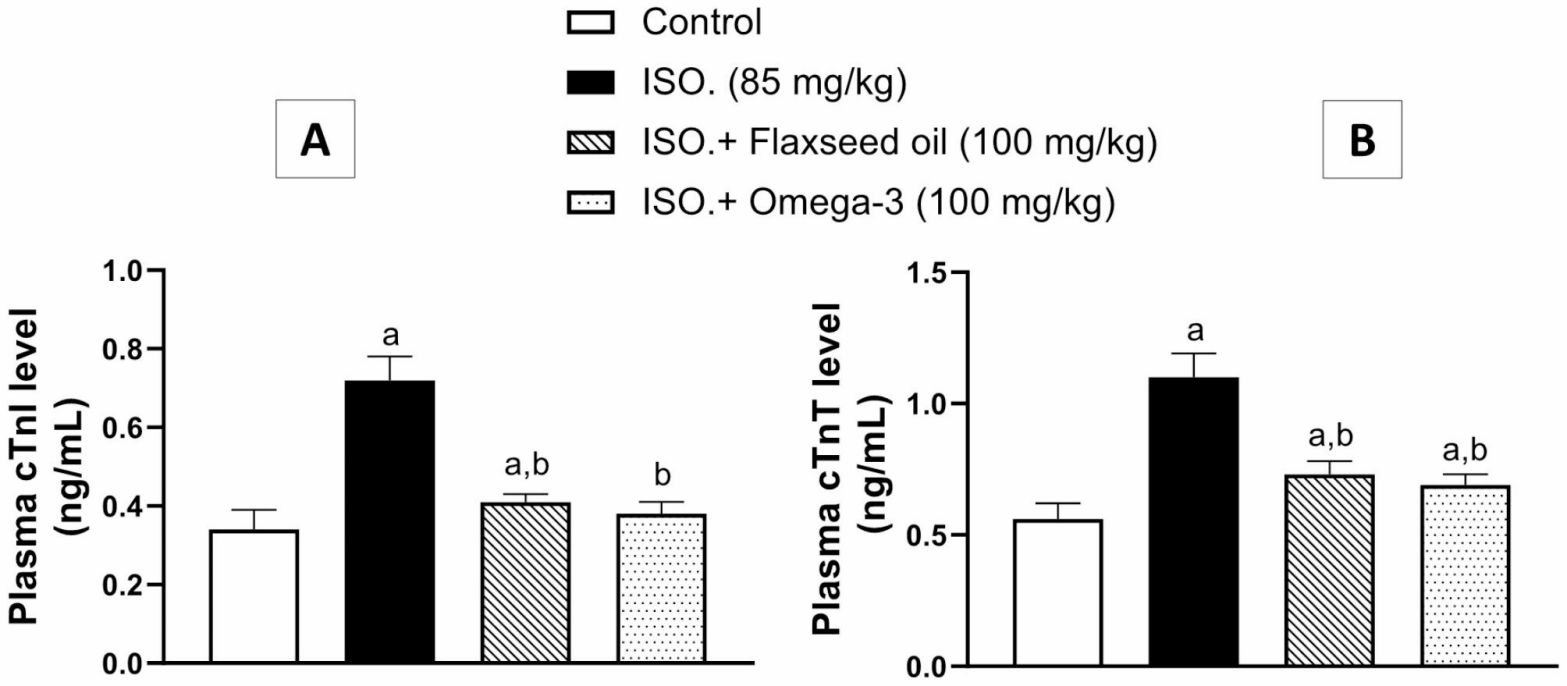
Effect of 28-day pretreatment with flaxseed oil on: [**A**] cTnI and [**B**] cTnT in isoproterenol-induced cardiac remodeling in rats. Data are represented as mean ± S.D. n = 8. Statistical analysis was performed utilizing one-way ANOVA followed by Tukey’s post-hoc test. The level of significance was set at p < 0.01. (**a**) is significantly different from the corresponding control group; (**b**) is significantly different from the corresponding isoproterenol-challenged group. cTnI: cardiac Troponin I; cTnT: cardiac Troponin T

The plasma levels of cardiac markers “cTnI and cTnT” were assessed, as shown in Fig. [3 A & 3B]; respectively. Based on the current findings, ISO administration (group II) significantly raised (*p* < 0.01) plasma levels of both markers by two folds, in comparison to the corresponding control groups. Moreover, pretreatment with flaxseed oil (group III) caused a substantial reduction (*p* < 0.01) in plasma cTnI and cTn T levels by about 43% and 34%, respectively, when compared to ISO group (II). Likewise, the levels of cTnI and cTnT significantly dropped by standard pretreatment with omega-3 (group IV) by 47% and 37%, respectively (*p* < 0.01), when related to ISO group (II).

**Fig. 4:**
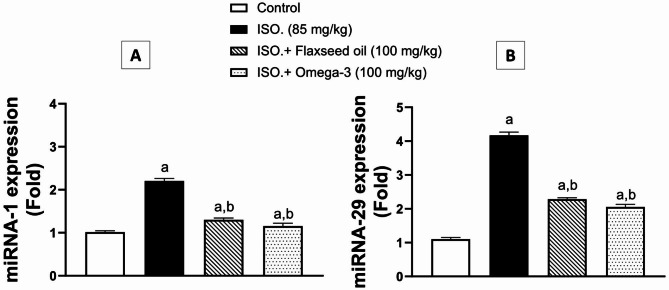
Effect of 28-day pretreatment with flaxseed oil on the expression of: [**A**] miRNA-1 and [**B**] miRNA-29 in isoproterenol-induced cardiac remodeling in rats. Data are represented as mean ± S.D. n = 3. Statistical analysis was performed utilizing one-way ANOVA followed by Tukey’s post-hoc test. The level of significance was set at p < 0.01. (**a**) is significantly different from the corresponding control group; (**b**) is significantly different from the corresponding isoproterenol-challenged group

As indicated in Fig. (4 A & 4B), the gene expressions of miRNA-1 and miRNA-29 were significantly intensified by about two and four-fold, respectively, upon ISO administration (group II) in contrast to the corresponding control group. However, group III – pretreated with Flaxseed - showed significant declines (*p* < 0.01) in miRNA-1 and miRNA-29 gene expressions, by 41% and 45%, respectively, as compared to ISO group (II). The effect of omega-3 pretreatment (group IV) was in harmony with that of flaxseed oil, where miRNA-1 and miRNA-29 expressions decreased significantly (*p* < 0.01) by 47% and 51%, respectively, compared to the ISO group (II).

## Discussion

In the recent decade, essential fatty acids (EFAs) become the primary focus of scientific research in the field of cardiac medicine [41]. Despite this finding progress, the molecular mechanisms and the exact physiological ability of dietary essential fatty acids that prevent associated cardiovascular diseases are still unanswered [42]. The current study describes how flaxseed oil-rich ALA could have a cardiac protective effect against cardiac remodeling at the molecular and genetic levels. The chemical analysis revealed that alpha-linolenic acid represents 9.8% of extracted crude oil and 4.8% of total flaxseed weight. ALA (18:3n-3) is an 18-carbon atom carboxylic acid with three cis double bonds and is considered an essential fatty acid indispensable to the human body. It can convert into eicosapentaenoic acid and docosahexaenoic acid in the biological system. However, this conversion is limited and affected by many factors such as gender, dose, and disease [43].

The proper analysis of the cell signaling involved in cardiac remodeling may assist in the development of novel therapeutic approaches for the treatment of heart failure and the mitigation of cardiac repercussions [44]. Cardiac hypertrophy, fibrosis, metabolic abnormalities, and mitochondrial dysfunction are part of an integrated signaling network that characterizes myocardial remodeling. The hypertrophic genetic program includes the upregulation of BNP and ANP signaling molecules [45]. Moreover, they are commonly used as key indicators for the clinical diagnosis of heart failure (HF) and cardiac dysfunction [46, 47]. The cTnT and cTnI are also considered specific markers of predicting cardiomyopathy progression end-stage phase [48]. The pathological process of hypertrophic cardiomyopathy (HCM)depends critically on microRNas (miRNAs), a class of noncoding RNA molecules. Therefore, miRNAs associated with HCM are considered a potential new therapeutic target [49]. The current study revealed that flaxseed oil inhibits BNP, NT-pro-BNP, cTnI, and cTnT and decreases the levels of miRNA-1 and miRNA-29b gene expression which indicates that the flaxseed oil is a good candidate to reduce apoptosis and cardiac fibrosis.

The ongoing myocardial inflammation leads to cardiac remodeling that could develop into heart failure. Chronic inflammation lowers ATP and phosphocreatine concentrations and impairs fatty acid oxidation and mitochondrial glucose metabolism [50]. As a result, this acidogenic process provides anaerobic energy. Lp-PLA2 contributes to vascular inflammation-related diseases and mediates macrophage migration, which in consequence plays a crucial role in hypertensive cardiac remodeling [51]. The increased levels of endothelin-1 (ET-1) and Lp-PLA2 have been linked to different heart conditions, including interferon-induced chronic active myocarditis and cardiomyopathy [52, 53]. The group of isopropanol-treated rats showed an increase in, Lp-PLA2, plasma levels, which indicates that isopropanol induces cardiac inflammation. It is tempting to speculate that these mediators’ increased expression is accessible to myocardial toxicity. Our study identifies a novel anti-inflammatory and anti-cardiac fibrosis role of flaxseed oil in Lp-PLA2 inhibition.

Interstitial and perivascular fibrosis, a condition brought on by the interaction of inflammation and apoptosis, frequently surrounds hypertrophy. Indeed, fibrosis reduces contractility and interferes with the heart’s chemoelectrical conductance, resulting in arrhythmias, local micro-fibrillations, and ineffective contraction [54]. Fibrosis downregulation of MMP inhibitors demands increased production of matrix metalloproteinases (MMPs) [55]. On the contrary, the flaxseed oil intake decreases the levels of MMP2 in the group animal model.

Aging is an inevitable process of life that is characterized by a steady decrease in tissue function and physiology [56]. Aging and sex hormone deprivation is considered primary factor of cardiac remodeling, where the heart “shrinking”, smaller ventricular volumes, and changes in left ventricle (LV) morphology have been reported [53]. Heterochromatin is a cell dark-staining, which contains a higher local concentration of protein and nucleic acid [57]. The crucial role of heterochromatin is controlling gene expression and protecting the genome from DNA damage, previous evidence supported the heterochromatin loss model during the aging process [58]. Topoisomerases are unique enzymes that regulate genome functions, including DNA replication and transcription [59]. These enzymes aid in resolving DNA topological (property and geometry) as they can decatenate intertangled DNA produced by transcription, replication, recombination, and chromosomal condensation and segregation, as well as relax supercoiled DNA [58].

According to early studies examining Top 2 A and Top 2B’s differential expression, Kondapi, and colleagues discovered that Topo 2B lower expression is associated with aging in adult rats [60]. More particular, the cerebellum and cerebellar area showed a decrease in protein expression. This was further supported by the observation that Top 2B activity declined with age in sheep neural cell preparation [61, 62]. Hence, the investigation of Topo 2B levels in ISO-treated rats was important. Our results showed a reversible correlation between Topo 2B and cardiac inflammation while plasma Topo 2B levels were significantly decreased in ISO-treated rats. The administration of flaxseed oil increased the level of plasma Topo 2B with subsequent inhibition of inflammation and improvement of cardiac tissue.

## Conclusion

The current study has provided experimental evidence that standardized flaxseed oil was effective in protecting against isoproterenol-induced cardiac remodeling and improving cardiac function. The findings have clarified the molecular mechanism of flaxseed oil as a potential cardioprotective drug candidate for reducing the incidence of cardiovascular risk factors. The study also observed that the flaxseed oil downregulated several biomarkers, including BNP, NT-pro-BNP, endothelin-1, Lp-PLA2, and MMP2, as well as cTnI and cTn plasma levels. Additionally, the oil upregulated Topo 2B and down-regulated the expression of miRNA-1 and miRNA-29b genes. Further research is recommended to investigate the bioavailability, dose, gender, and other factors that may impact the pharmacokinetics of flaxseed-rich ALA oil.

### Electronic supplementary material

Below is the link to the electronic supplementary material.

Supplementary Material 1

## Data Availability

The datasets used and/or analyzed during the current study available from the corresponding author on reasonable request.

## Abbreviations

ALA: Alpha-linolenic acid
BNP: Brain natriuretic peptide
cTnI: Cardiac troponin I
cTnT: Cardiac troponin T
CVDs: Cardiovascular disease
ELISA: Enzyme-linked immunoassay
ET-1: Endothelin 1
GC-MS: Gas chromatography-mass spectrometry
HFD: Heart failure disease
HMGCR: Hydroxymethylglutaryl coenzyme A reductase
IL-6: Interleukin-6
ISO: Isoproterenol
Lp-PLA2: Lipoprotein-Associated Phospholipase A2
LTB5: Leukotrienes B5
MCP-1: Monocyte chemoattractant protein-1
MMP9: Matrix metalloproteinase 9
NADPH oxidase: Nicotinamide adenine dinucleotide phosphate oxidases
NT-pro-BNP: N-terminal pro?B-type natriuretic peptide
PGE3: Prostaglandin E3
PPARα: Peroxisome proliferator-activated receptor alpha
RT-PCR: Reverse transcription polymerase chain reaction
SREBPs: Sterol regulatory element binding proteins
TNF: Tumor necrosis factor
Top2a: DNA Topoisomerase II Alpha
Top2b: Topoisomerase 2 beta
VCAM-1: Vascular cell adhesion molecule 1
VSMCs: Vascular smooth muscle cells
WHO: World Health Organization
