# Supplementary material for: Flaxseed oil fraction reverses cardiac remodeling at a molecular level: improves cardiac function, decreases apoptosis, and suppresses miRNA-29b and miRNA 1 gene expression
Source: BMC Complement Med Ther. 2024 Jan 2;24:6. doi: 10.1186/s12906-023-04319-8 (PMC10759513; doi:10.1186/s12906-023-04319-8)
Supplement: Supplementary file 1 — Supplementary Material 1 [file 12906_2023_4319_MOESM1_ESM.docx]

**Supplementary File**

**Flaxseed oil fraction reverse cardiac remodeling at molecular level: Improves cardiac function, decreases apoptosis, and suppresses miRNA-29b and miRNA 1 gene expression**

Sylvia A Boshra ^1^*****, Jilan A. Nazeam ^2^*, Ahmed Esmat^3^

^1^Biochemistry Department, Faculty of Pharmacy, October 6 University, *^6^th of October City 12511*, Egypt.

^2^Pharmacognosy Department, Faculty of Pharmacy, October 6 University, *^6^th of October City 12511*, Egypt

^3^Department of Pharmacology and Toxicology, Faculty of Pharmacy, Ain Shams University, *Cairo, 11566*, Egypt

**Table I**

GC-MS of purified flaxseed oil fraction.

| Peak | R. Time | Area% | Name | Base m/z |
| --- | --- | --- | --- | --- |
| 1 | 6.246 | 0.01 | Ethanol, 2-butoxy- | 57.10 |
| 2 | 9.019 | 0.02 | Furan, 2-pentyl- | 81.05 |
| 3 | 9.237 | 0.02 | 3-Methyl-but-2-enoic acid, 1,7,7-trimethyl-bicyclo [2.2.1] hept-2-yl ester | 83.10 |
| 4 | 13.112 | 0.01 | Octanoic acid, methyl ester | 74.10 |
| 5 | 18.459 | 0.01 | Rhodium, [1,2-bis(eta.2-ethenyl)-4-ethenylcyclohexane]di-.mu.-chlorodi- | 121.10 |
| 6 | 18.523 | 0.02 | Furan, 2-hexyl- | 81.05 |
| 7 | 20.894 | 0.01 | n-Caprylic acid isobutyl ester | 127.15 |
| 8 | 21.471 | 0.03 | Nonanoic acid, 9-oxo-, methyl ester | 111.15 |
| 9 | 22.743 | 0.07 | Cycloheptanone, 3-butyl- | 111.15 |
| 10 | 24.584 | 0.02 | Nonanedioic acid, dimethyl ester | 111.15 |
| 11 | 26.557 | 0.03 | Imidazole-5-pentanoic acid | 95.10 |
| 12 | 27.449 | 0.05 | 2-n-Heptylfuran | 81.05 |
| 13 | 28.156 | 0.05 | Azelaaldehydic acid, butyl ester | 109.15 |
| 14 | 29.276 | 0.01 | Heptadecanoic acid, 16-methyl-, methyl ester | 87.10 |
| 15 | 30.011 | 0.02 | Tetradecanoic acid | 129.15 |
| 16 | 30.748 | 0.02 | Nonanedioic acid, dimethyl ester | 125.15 |
| 17 | 32.569 | 0.02 | Imidazole-5-pentanoic acid | 95.10 |
| 18 | 33.638 | 1.50 | Hexadecanoic acid, methyl ester | 87.10 |
| 19 | 34.493 | 4.02 | n-Hexadecanoic acid | 73.10 |
| 20 | 35.020 | 0.01 | Decanoic acid, 2,4,6-trimethyl-, methyl ester | 88.10 |
| 21 | 36.850 | 2.07 | 9,12-Octadecadienoic acid (Z,Z)-, methyl ester | 81.10 |
| 22 | 36.933 | 2.94 | 9,12,15-Octadecatrienoic acid, methyl ester, (Z,Z,Z)- | 79.10 |
| 23 | 37.053 | 4.45 | Elaidic acid | 97.15 |
| 24 | 37.589 | 2.55 | Methyl stearate | 87.10 |
| 25 | 37.995 | 24.69 | Alpha-linolenic acid | 79.10 |
| 26 | 38.095 | 10.57 | Oleic Acid | 83.15 |
| 27 | 38.419 | 2.63 | Octadecanoic acid | 43.10 |
| 28 | 38.734 | 2.71 | Hexadecanoic acid, butyl ester | 56.10 |
| 29 | 38.860 | 0.05 | Heptadecanoic acid, ethyl ester | 88.10 |
| 30 | 39.985 | 0.06 | Ether, (2-ethyl-1-cyclodecen-1-yl)methyl methyl | 125.15 |
| 31 | 40.138 | 0.07 | (Z,Z,Z)-6,9,15-Octadecatrienoic acid methyl ester | 95.15 |
| 32 | 40.216 | 0.09 | 9-Octadecenoic acid, 12-hydroxy-, methyl ester, (Z)- | 95.10 |
| 33 | 40.685 | 0.05 | cis-Methyl 11-eicosenoate | 97.15 |
| 34 | 40.874 | 0.27 | 9,12-Octadecadien-1-ol, (Z,Z)- | 81.10 |
| 35 | 40.951 | 0.42 | 9,12,15-Octadecatrienoic acid, ethyl ester, (Z,Z,Z)- | 79.10 |
| 36 | 41.026 | 0.51 | Octyl cis-vaccenate | 97.15 |
| 37 | 41.140 | 0.03 | 5,5,8a-Trimethyldecalin-1-one | 111.15 |
| 38 | 41.209 | 0.07 | Eicosanoic acid, methyl ester | 87.10 |
| 39 | 41.274 | 0.05 | 13-Docosenamide, (Z)- | 72.10 |
| 40 | 41.598 | 3.22 | 9-Octadecen-1-ol, acetate, (Z)- | 81.10 |
| 41 | 41.689 | 6.05 | n-Propyl linolenate | 79.10 |
| 42 | 41.771 | 7.05 | 2,3-Dihydroxypropyl elaidate | 83.10 |
| 43 | 41.846 | 0.29 | cis-9-Octadecenoic acid, propyl ester | 97.15 |
| 44 | 42.236 | 1.89 | Octadecanoic acid, butyl ester | 56.10 |
| 45 | 42.689 | 0.03 | Cedrol | 95.10 |
| 46 | 43.352 | 0.01 | Glycidyl (Z)-9-nonadecenoate | 129.10 |
| 47 | 43.780 | 0.97 | Hexadecanoic acid, 2-hydroxy-1-(hydroxymethyl)ethyl ester | 98.10 |
| 48 | 44.063 | 0.06 | 6-Octadecenoic acid, methyl ester, (Z)- | 97.15 |
| 49 | 44.407 | 0.18 | Bis(2-ethylhexyl) phthalate | 149.05 |
| 50 | 44.520 | 0.11 | Heptadecanoic acid, 16-methyl-, methyl ester | 87.10 |
| 51 | 44.606 | 0.09 | 4H-Cyclopentacycloocten-4-one, decahydro- | 95.10 |
| 52 | 44.754 | 0.05 | 2-Buten-1-ol, 2-ethyl-4-(2,2,3-trimethyl-3-cyclopenten-1-yl)- | 121.15 |
| 53 | 45.000 | 0.07 | cis-13-Eicosenoic acid | 97.15 |
| 54 | 45.454 | 0.12 | Arachidic acid, butyl ester | 56.10 |
| 55 | 46.641 | 9.04 | [Glycerol oleate](https://www.chembk.com/en/chem/Glycerol%20oleate) | 98.15 |
| 56 | 46.722 | 0.98 | E,E,Z-1,3,12-Nonadecatriene-5,14-diol | 95.15 |
| 57 | 47.011 | 0.64 | Hexadecanedioic acid, dimethyl ester | 98.15 |
| 58 | 47.145 | 0.09 | Octadecanoic acid, 2,3-dihydroxypropyl ester | 98.15 |
| 59 | 47.604 | 0.05 | Tetracosanoic acid, methyl ester | 87.10 |
| 60 | 48.062 | 0.11 | Erucic acid | 97.15 |
| 61 | 48.460 | 0.06 | Docosanoic acid | 56.10 |
| 62 | 49.033 | 0.02 | 2,2,4-Trimethyl-3-(3,8,12,16-tetramethyl-heptadeca-3,7,11,15-tetraenyl)-cyclohexanol | 81.10 |
| 63 | 49.621 | 0.03 | E,E,Z-1,3,12-Nonadecatriene-5,14-diol | 95.15 |
| 64 | 49.996 | 0.02 | 1-Heptacosanol | 97.15 |
| 65 | 50.467 | 0.02 | Heptadecanoic acid, 16-methyl-, methyl ester | 87.10 |
| 66 | 51.459 | 0.02 | (-)-Globulol | 81.10 |
| 67 | 52.675 | 0.02 | 5-.alpha.-Androst-2-en-17-.beta.-ol, 17-methyl- | 105.10 |
| 68 | 53.383 | 0.03 | Andrographolide | 109.15 |
| 69 | 54.152 | 0.07 | Retinoic acid, methyl ester | 107.15 |
| 70 | 54.325 | 1.40 | Ergost-5-en-3-ol, (3.beta.)- | 107.10 |
| 71 | 54.788 | 0.48 | Humulenol-II | 83.15 |
| 72 | 55.414 | 0.03 | Andrographolide | 105.10 |
| 73 | 55.765 | 2.50 | gamma.-Sitosterol | 107.10 |
| 74 | 55.939 | 0.18 | Cholest-5-en-3-ol, 24-propylidene-, (3.beta.)- | 105.10 |
| 75 | 56.238 | 0.20 | 6.beta.Bicyclo[4.3.0]nonane,5.beta.-iodomethyl-1.beta.-isopropenyl-4.alpha.,5.alpha.- | 109.15 |
| 76 | 56.601 | 0.02 | 6.beta.Bicyclo[4.3.0]nonane,5.beta.-iodomethyl-1.beta.-isopropenyl-4.alpha.,5.alpha.- | 121.15 |
| 77 | 56.954 | 2.27 | 6.beta.Bicyclo[4.3.0]nonane,5.beta.-iodomethyl-1.beta.-isopropenyl-4.alpha.,5.alpha.- | 95.10 |
| 78 | 57.344 | 0.01 | Azulene, 1,2,3,3a,4,5,6,7-octahydro-1,4-dimethyl-7-(1-methylethenyl)-, [1R-(1. alpha) | 107.10 |
| 79 | 57.936 | 0.02 | Pregn-4-ene-3,20-dione, (9. beta.,10.alpha.)- | 124.10 |
| 80 | 58.060 | 0.03 | 6.beta. Bicyclo [4.3.0]nonane, 5.beta.-iodomethyl-1.beta.-isopropenyl-4.alpha.,5.alpha.- | 95.15 |
